# The Key Ingredient Acacetin in Weishu Decoction Alleviates Gastrointestinal Motility Disorder Based on Network Pharmacology Analysis

**DOI:** 10.1155/2021/5265444

**Published:** 2021-09-21

**Authors:** Xuan Guo, Yin Xu, Hua-liang Tan, Xiao-juan Wang, Lin Xiao

**Affiliations:** ^1^Institute of Traditional Chinese Medicine, Hunan University of Traditional Chinese Medicine, Changsha 410208, China; ^2^Department of Internal Medicine, The First Affiliated Hospital of Hunan University of Traditional Chinese Medicine, Changsha 410007, China; ^3^Hunan University of Chinese medicine, 300 bachelor's road, Yuelu District, Changsha City, Hunan Province, China

## Abstract

**Background:**

Gastrointestinal motility disorder is a common gastrointestinal disease, which seriously affects life quality. Traditional Chinese medicine (TCM) has been widely used as an alternative therapy for gastrointestinal motility disorder. Acacetin is a natural flavonoid compound that has antioxidant and anti-inflammatory, antidepressant, and anticancer properties. However, the efficacy of Acacetin in the treatment of gastrointestinal motility disorders has not been studied. Our aim was to investigate the mechanism of Acacetin-alleviated gastrointestinal motility disorder and its efficacy based on network pharmacology.

**Methods:**

We performed network pharmacology to predict the active components, match Weishu decoction (WSD) targets in gastrointestinal motility disorders, and investigate its potential pharmacological mechanisms. We performed the GO and KEGG enrichment analysis. *In vivo*, we investigated the effects of Acacetin in the gastrointestinal motility disorder model.

**Results:**

Based on network pharmacological method, the key active ingredient of WSD was identified as Acacetin, and the enrichment signaling pathway was the PI3K-AKT signaling pathway. Acacetin and Mosapride accelerated gastric emptying time, reduced gastric remnant rate, and increased small intestinal propulsion rate. The levels of GAS and MTL were increased after using Acacetin. These results indicated that Acacetin could improve gastrointestinal motility disorders. Among them, high-dose Acacetin showed a better effect. Acacetin could regulate protein and lipid metabolism in mice with gastrointestinal motility disorder. Furthermore, Acacetin could modulate gastrointestinal inflammation and apoptosis. The detection of the PI3K-AKT signaling pathway-related proteins showed that Acacetin improved gastrointestinal motility disorder by inhibiting the activation of the PI3K-AKT signaling pathway.

**Conclusion:**

The key ingredient Acacetin in WSD could alleviate gastrointestinal motility disorder by inhibiting the activation of the PI3K-AKT signaling pathway based on network pharmacology analysis. The efficacy and safety of Acacetin treatment provide strong experimental support for the clinical treatment of gastrointestinal motility disorder.

## 1. Introduction

Gastrointestinal motility disorder is a common gastrointestinal disease that seriously affects the quality of life and socioeconomic costs [1]. It is characterized by abnormal motor, sensory, or secretory functions that alter the intestinal function and lead to a significant disease burden [2]. Millions of patients worldwide suffer from gastrointestinal motility disorders, which include debilitating symptoms such as chronic nausea and vomiting [3]. The most severe gastrointestinal motility disorders may directly lead to intestinal failure [4]. It has been reported that up to 80% of critically ill patients suffer from gastrointestinal motility disorders. In critically ill patients, gastrointestinal dysfunction or gastrointestinal failure is associated with increased morbidity and mortality [5]. In addition to movement disorders, the factors leading to the decreased visceral perceptual threshold are common in the pathogenesis of functional gastrointestinal diseases [6]. However, the pathogenesis of gastrointestinal motility disorder remains unclear and may be closely associated with gastrointestinal hypersensitivity, impaired food intake regulation, delayed gastric emptying, lipoprotein metabolism, and neuropsychological factors. Although many drugs have been developed to treat gastrointestinal motility disorders, very few drugs are currently available.

Traditional Chinese medicine (TCM) is the precious treasure of the Chinese nation. Thousands of years of practical experience has made significant contributions to preventing and treating human diseases [7]. At present, TCM has been widely used as an alternative therapy for gastrointestinal motility disorders. Weishu decoction (WSD) consists of Radix Bupleurum 10 g, Cyperus rotundus 10 g, unripe bitter orange 10 g, Rhizoma Atractylodes 10 g, White Peony 10 g, Fructus Toosendan 6 g, Rhizoma Corydalis 10 g, Inula flowerlog 10 g, and Charred medicated leaven 10 g, which can alleviate gastrointestinal motility disorder [8]. However, the exact mechanism of the WSD effect on gastrointestinal motility disorder remains unclear.

With the vigorous development of network pharmacology in recent years, a new approach to finding therapeutic drugs has emerged [9]. Network pharmacology is the combined analysis of TCM preparations that provides a powerful tool for establishing a “complex protein/gene-disease” network and revealing the regulation principle of small molecules [10]. Its core theory is a new holistic and systematic “network goal” method [11]. The holistic philosophy of TCM has much in common with the core ideas of emerging network pharmacology and network biology. It can systematically overcome the needs of complex diseases such as cancer [12]. Therefore, this study was based on network pharmacology to study the mechanism of WSD's key active ingredient on gastrointestinal motility disorder.

## 2. Material and Methods

### 2.1. Disease Target Identification and Network Establishment

The TCMSP online database (https://tcmspw.com/tcmsp.php) [13] was used to retrieve the ADME parameter information of the components of WSD, and the components with drug − likeness (DL) ≥ 0.18 [14] were included to predict the component targets. The included compounds were identified by the SwissTargetPrediction database (http://www.swisstargetprediction.ch/) [15], and the target with probability greater than 0 was finally contained. Taking “Gastrointestinal motility disorders” and “disorders of gastrointestinal motility (DGIM)” as keywords, human genes were searched in the GeneCards database (https://www.genecards.org/) [16], NCBI gene database (https://www.ncbi.nlm.nih.gov/) [17], and OMIM database (https://www.omim.org/) [18]. Among them, GeneCards data filtered the median value of the relevance score to obtain more relevant targets. In R software analysis, all drug-protein targets and disease-related proteins were classified into two independent groups. The screened drug targets and disease targets were input into the Venn 2.1. The common targets of drug diseases were input into the String database (https://string-db.org/cgi/input.pl) to construct the protein-protein interaction (PPI) network [19]. The species was set as “*Homo sapiens*,” and the credibility was set as >0.9. In order to better understand the complex interaction relationship between components, diseases, and corresponding targets, a component-disease-target network diagram was constructed based on the included components, therapeutic diseases, and targets and was imported into Cytoscape 3.8.0 to draw the network diagram. Then, the topology analysis was carried out by NetworkAnalyzer [20]. Genes with a score greater than average were selected as key targets by degree sorting. In this study, key genes were screened through the MCODE analysis [21].

### 2.2. Bioinformatic Annotation

GO biological process (BP), molecular function (MF), and cell component (CC) enrichment were carried out on the common targets of drug diseases, and the items with corrected *P* ≤ 0.05 were screened using the String database. The ClusterProfiler, enrichplot, and ggplot2 packages were installed, and the R 3.6.3 software was used to make histograms and bubble plots. KEGG pathway enrichment analysis was carried out on common targets of drug diseases, and the items with corrected *P* ≤ 0.05 were also screened using the String database.

### 2.3. Establishment of DGIM Model

The adult SPF C57BL/6 male mice were raised in a temperature- and humidity-controlled animal facility on a 12-hour light/dark cycle. The mice were randomly divided into 9 groups with 12 mice in each group. They were divided into the control group (CG), the model group (MG), the low-dose Acacetin group (LAG), the middle-dose Acacetin group (MAG), the high-dose Acacetin group (HAG), the Mosapride group (MPG) (positive control). Acacetin was purchased from Selleck Chemicals (#S5318, Houston, Texas, USA), dissolved in dimethyl sulfoxide (DMSO), and stored at -20°C. The model of gastrointestinal motility disorder induced by atropine [22]. Acacetin was orally administered 10, 25, and 50 mg/kg for LAG, MAG, and HAG, respectively [23], and Mosapride was orally administered 1.37 mg/kg for MPG [24]. Distilled water (10 mL/kg) was given to the CG and MG. All treatment groups were treated once a day for 14 days. On the 14th day, after taking the drugs for 1 h, except the CG, the other groups were injected with atropine 1.5 mg/kg. After 30 min, all mice were orally administered 0.5 mL of semisolid paste (0.2% CMC-Na and 5% carbon), and 30 min later, blood was collected.

### 2.4. Gastric Remnant Rate and Small Intestinal Propulsion Rate

The mice were orally administered 0.5 mL of semisolid paste and were killed 30 min later. The difference between total gastric weight and net gastric weight was the residual weight in the stomach, and the percentage of residual weight in the stomach and the weight of semisolid paste was the gastric remnant rate (%). The small intestine of mouse was put on the plane without traction, and the total length from the pylorus to the ileocecal area and the distance from the pylorus to the front end of carbon were measured. The percentage of the advancing length of semisolid paste and the total length of the small intestine was the small intestinal propulsion rate [25].

### 2.5. Enzyme Linked Immunosorbent Assay (ELISA)

ELISA was performed to detect serum gastrin (GAS) and motilin (MTL), serum protein metabolism indexes prealbumin (PAB), ceruloplasmin (CER) and transferrin (TRF), and lipid metabolism indexes triglyceride (TG), low-density lipoprotein cholesterol (LDL-C), high-density lipoprotein cholesterol (HDL-C), total cholesterol (TC), and inflammatory factors TNF-*α*, IL-1*β*, and IL-6 levels. The GAS, MTL, PAB, CER, TRF, TNF-*α*, IL-1*β*, and IL-6 were detected by GAS (#CSB-E12743r, CUSABIO, China), MTL (#CSB-E08208r, CUSABIO, China), PAB (MLBIO, China), CER (MLBIO, China), TRF (#CSB-E12723r, CUSABIO, China), TNF-*α* (#CSB-E11987r, CUSABIO, China), IL-1*β* (#CSB-E08055r, CUSABIO, China), and IL-6 (#CSB-E04640r, CUSABIO, China) ELISA kit according to the instructions. The concentration of GAS, MTL, PAB, CER, TRF, TNF-*α*, IL-1*β*, and IL-6 was calculated using the Bio-Tek microplate analyzer (MB-530, HEAES, China) by forming a standard curve based on the provided values. The concentration of TG, LDL-C, HDL-C, and TC were detected by the TG (#A110-2-1, Nanjing Jiancheng Bioengineering Institute, China), LDL-C (#A113-2-1, Nanjing Jiancheng Bioengineering Institute, China), HDL-C (#A112-2-1, Nanjing Jiancheng Bioengineering Institute, China), and TC (#A111-2-1, Nanjing Jiancheng Bioengineering Institute, China) kit according to the instructions.

### 2.6. Quantitative Real-Time PCR (qRT-PCR)

The relative expression levels of Bcl-2, Bax, Caspase-12, TNF-*α*, IL-1*β*, and IL-6 in the gastric antrum tissues were detected by qRT-PCR. Total RNA was extracted by Trizol methods; RNA was reversely transcribed into cDNAs in accordance with the instruction of a reverse transcription kit (#4368814, Invitrogen, USA). SYBR Green qPCR mix (Invitrogen) was performed to test gene relative expression in ABI 7900 system. The relative level of the gene was calculated by 2^−ΔΔCt^ method with *β*-actin as the internal gene. The primer sequences used in this study are shown in Table 1.

### 2.7. Western Blot

RIPA lysis buffer (#P0013B, Beyotime) was applied to extract the total protein from the gastric antrum tissues. The protein was mixed with the SDS-PAGE loading buffer (#MB2479, Meilunbio) for 5 min in boiling water at 100°C. The proteins were separated by gel electrophoresis and transferred to the PVDF membrane. Then, they were sealed with 5% skim milk solution for 2 h at room temperature, and incubated with diluted primary antibodies TNF-*α* (ab255275, 1: 1000, Abcam, UK), IL-1*β* (ab9722, 0.2 *μ*g/mL, Abcam, UK), IL-6 (ab233706, 1: 1000, Abcam, UK), Bax (ab32503, 1: 5000, Abcam, UK), Caspase-12 (ab62463, 1 *μ*g/mL, Abcam, UK), Bcl-2 (ab182858, 1: 2000, Abcam, UK), PI3K (ab191606, 1: 5000, Abcam, UK), p-PI3K(ab182651, 1: 800, Abcam, UK), AKT (10176-2-AP, 1: 1000, Proteintech, USA), p-AKT (66444-1-Ig, 1: 5000, Proteintech, USA), and *β*-actin (66009-1-Ig, 1: 5000, Proteintech, USA) at room temperature for 90 min. The secondary antibody HRP goat anti-mouse IgG (SA00001-1, 1: 5000, Proteintech, USA) or HRP goat anti-Rabbit IgG (SA00001-2, 1: 6000, Proteintech, USA) was incubated with the membrane at room temperature for 90 min. The protein bands were detected by the Chemiscope6100 system (Clinx Co., Ltd, Shanghai, China). The integrated density of protein bands was determined by the Quantity One 4.6.2 software and corrected by subtracting the measured integrated density with the background integrated density. *β*-Actin was used as the internal reference for detecting relative expression levels.

### 2.8. Immunohistochemistry (IHC)

The expressions of PI3K, p-PI3K, AKT, and p-AKT were detected by IHC in the gastric antrum tissues of different groups. The slices were roasted at 60°C for 12 h. Then, the slices were dewaxed to water and heated to repair the antigen. 1% periodic acid was added, and the endogenous enzyme was inactivated for 10 min at room temperature. The PI3K (ab151549, 1: 100, Abcam, UK), p-PI3K (ab182651, 1: 100, Abcam, UK), AKT (10176-2-AP, 1: 100, Proteintech, USA), and p-AKT (66444-1-Ig, 1: 100, Proteintech, USA) primary antibodies were incubated overnight at 4°C. The secondary antibody was incubated at 37°C for 30 min. DAB was used for color development, hematoxylin was restained for 10 min, washed with distilled water, and PBS returned to blue. All levels of alcohol were dehydrated for 5 min. After removal, it was placed in xylene for 10 min. Then, it was sealed with neutral gum and observed under the microscope. We selected the location of the gastric antrum, taking a 400-fold field of view. Image-pro-plus 6.0 analysis software was chosen for IOD analysis. The average optical density (positive area IOD under the field of view/tissue area under the field of view) was performed to indicate the relative expression of PI3K, p-PI3K, AKT, and p-AKT.

### 2.9. Statistical Analysis

Statistical analysis was performed using GraphPad 8.0 software, and three independent experimental data were expressed as mean ± standard deviation (SD). The unpaired *T*-test was used between the two groups conforming to the normal distribution. The one-way analysis of variance (ANOVA) was conducted among multiple groups, followed by Tukey's post hoc test. *P* < 0.05 was considered statistically significant.

## 3. Results

### 3.1. The Assumed Targets of WSD

In this study, the TCMSP online database (https://tcmspw.com/tcmsp.php) [13] was used to retrieve the ADME parameter information of the components of WSD, and the components with DL ≥ 0.18 [14] were included to predict the component targets. The included compounds were identified by the SwissTargetPrediction database (http://www.swisstargetprediction.ch/) [15], and the target with probability greater than 0 was finally contained. As shown in Supplementary Table 1, a total of 15 active components were included in WSD. We showed the average physicochemical value of each component, including the molecule weight (MW), the value of partition coefficient between octanol and water (ALogP), hydrogen-bond donors (Hdon), hydrogen-bond acceptors (Hacc), oral bioavailability (OB), blood brain barrier (BBB), DL, and fractional water accessible surface area of all atoms with negative partial charge (FASA-). Supplementary Table 2 showed a total of 460 target compounds in WSD. Taking “Gastrointestinal motility disorders” and “disorders of gastrointestinal motility (DGIM)” as keywords, human genes were searched in the GeneCards database (https://www.genecards.org/) [16], NCBI gene database (https://www.ncbi.nlm.nih.gov/) [17], and OMIM database (https://www.omim.org/) [18]. Among them, the GeneCards data filtered the median value of the relevance score to obtain more relevant targets. After searching, 2691 genes were found in the Genecards database, 3 genes were found in the NCBI database, and 618 targets were found in the OMIM database. After the combined deletion of these three databases, 3216 genes related to gastrointestinal motility disorders were obtained.

### 3.2. Network Analysis of Targets

**Figure 1:**
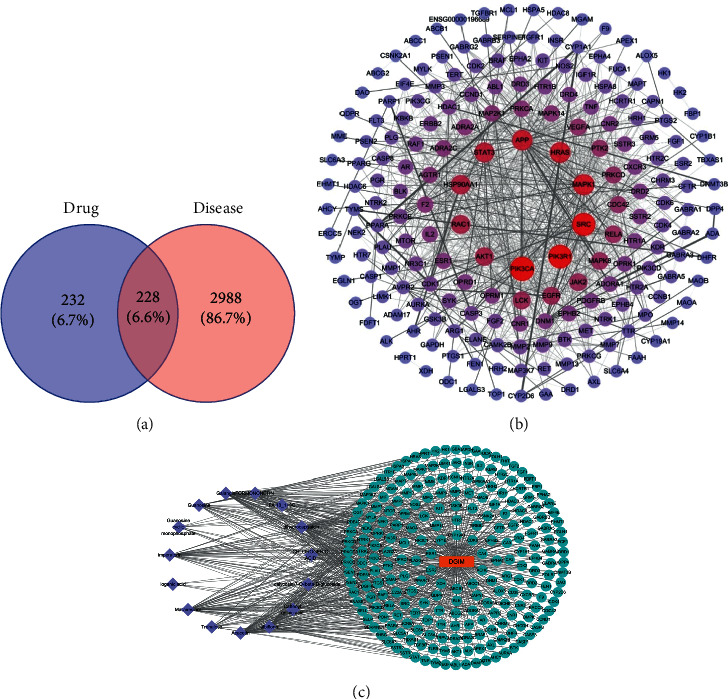
Network analysis of targets. (a) Venn diagram of screened drug targets and disease targets. (b) PPI network diagram of common targets for drug diseases. The color and size of the node were adjusted according to the degree value. The larger the color, the deeper the degree value; the thickness of the line, from thick to thin, indicated that the edge betweenness was from large to small. (c) Component-disease-target network diagram. Among them, the lavender circle was the active ingredient, green was the target of drug action on the disease, and orange was the disease.

In the R software analysis, all drug-protein targets and disease-related proteins were classified into the two independent groups. The screened drug targets and disease targets were input into the Venny 2.1. As shown in Figure 1(a), a total of 228 common targets were obtained from the Venny diagram of the screened drug targets and disease targets. The common targets of drug diseases were input into the String database (https://string-db.org/cgi/input.pl) [19] to construct the PPI network. The species was set as “*Homo sapiens*,” and the credibility was set as >0.9. There were 228 nodes and 1017 edges in the PPI network, and the average degree was 8.92.  Figure 1(b) showed the PPI network diagram of drug-disease common targets. At the same time, we constructed the component-disease-target network diagram (Figure 1(c)). To better understand the complex interaction relationship between components, diseases, and corresponding targets, a component-disease-target network diagram was imported into the Cytoscape 3.8.0 to draw the network diagram. Then, the topology analysis was carried out by the NetworkAnalyzer [20]. In our study, we found 15 active ingredients. The higher the degree value was, the more important the component was (Table 2). Among them, Acacetin had the highest degree. Therefore, Acacetin might be the key active ingredient in WSD.

### 3.3. Predicting Functional Enrichment Analysis for WSD

**Figure 2:**
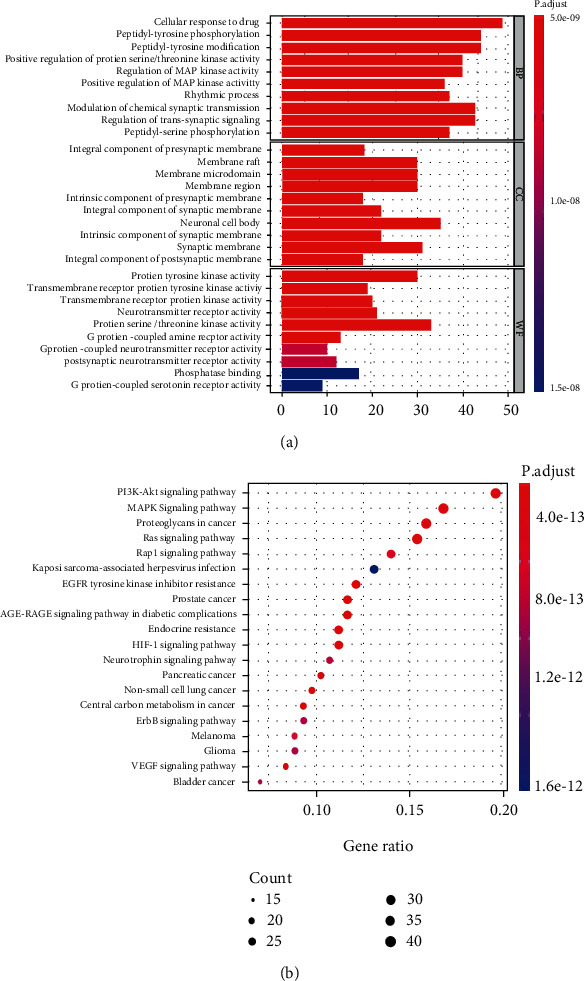
Bioinformatic analyzed of drug-disease intersection targets. (a) Gene ontology annotations. (b) KEGG annotation.

**Figure 3:**
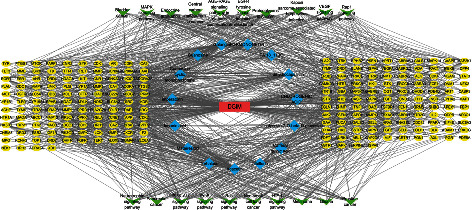
Component-disease-pathway-target network. Blue was the compound, yellow was the target of traditional Chinese medicine, and green was the top 20 pathways with the most significant enrichment.

The R package provided for GO annotation enrichment analysis, including the CC, MF, and BP analyses. GO annotation showed that the drug-disease crossover proteins were mainly related to the cellular response to drug, peptidyl-tyrosine phosphorylation, peptidyl-tyrosine modification of BP, the national cell body, synaptic membrane of CC, the protein serine/threonine kinase activity, and protein tyrosine kinase activity of MF (Figure 2(a)). In addition, KEGG enrichment analysis was closely associated with the PI3K-AKT signaling pathway, MAPK signaling pathway, and proteoglycans in cancer (Figure 2(b)). Then, we drew the network diagram of component-disease-pathway-target to more intuitively show the multicomponent-multitarget action characteristics of active ingredients of TCM in the treatment of gastrointestinal motility disorders. As shown in Figure 3, blue was the compound, including Acacetin, Galangin, dihydrocapsaicin, Matairesinol, albiflorin, and imperatorin. Yellow was the target of TCM, and green was the top 20 pathways with the most significant enrichment. Based on these predicted molecular mechanisms and the network analysis results, we designed *in vivo* experiments to test whether Acacetin could inhibit gastrointestinal inflammation and apoptosis from alleviating gastrointestinal motility disorders.

### 3.4. Acacetin Improved Gastrointestinal Motility in Mice

**Figure 4:**
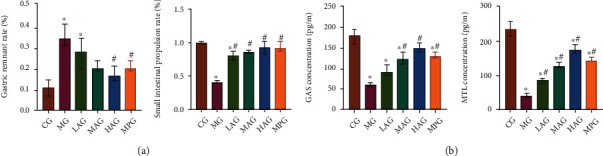
Acacetin improved gastrointestinal motility in mice. (a) Comparison of gastric remnant rate and small intestinal propulsion rate in each group. (b) The serum levels of GAS and MTL were detected by ELISA. CG: control group; MG: model group, LAG: low-dose Acacetin group; MAG: middle-dose Acacetin group; HAG: high-dose Acacetin group; MPG: Mosapride group; ^∗^*P* < 0.05 vs. CG; ^#^*P* < 0.05 vs. MG.

By constructing the gastrointestinal motility disorder model, we found that compared with the CG, gastric emptying was delayed, gastric remnant rate was increased, and small intestinal propulsion rate was decreased in the MG. Compared with the MG, Acacetin and Mosapride accelerated gastric emptying time, reduced gastric remnant rate, and increased small intestinal propulsion rate. These results revealed that Acacetin and Mosapride could improve gastrointestinal motility, and high-dose Acacetin had the better effect (Figure 4(a)). The results of ELISA showed that compared with the CG, the contents of MTL and GAS in the MG decreased significantly. Compared with the MG, the levels of GAS and MTL increased after using Acacetin and Mosapride (Figure 4(b)). These results indicated that Acacetin could regulate the contents of GAS and MTL in mice with gastrointestinal motility disorder, and the high-dose Acacetin had the better effect.

### 3.5. Acacetin Regulated Protein and Lipid Metabolism in Gastrointestinal Motility Disorder Mice

**Figure 5:**
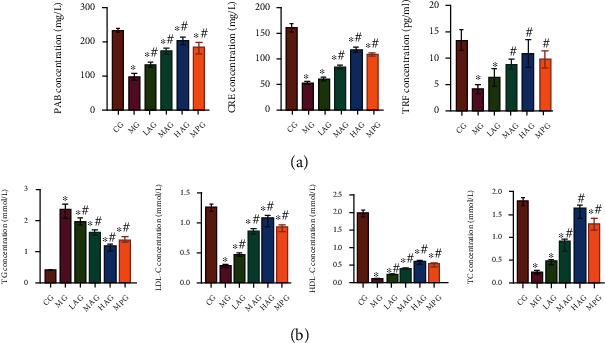
Acacetin regulated protein and lipid metabolism in gastrointestinal motility disorder mice. (a) ELISA was used to detect PAB, CER, and TRF levels. (b) The serum levels of TG, LDL-C, HDL-C, and TC were detected by ELISA. CG: control group; MG: model group; LAG: low-dose Acacetin group; MAG: middle-dose Acacetin group; HAG: high-dose Acacetin group; MPG: Mosapride group; ^∗^*P* < 0.05 vs. CG; ^#^*P* < 0.05 vs. MG.

To study whether the alleviating effect of Acacetin on gastrointestinal motility disorder was related to protein and lipid metabolism, we used ELISA to detect indicators related to protein metabolism and lipid metabolism. ELISA results showed that the levels of PAB, CER, and TRF were decreased in the MG compared with the CG. Compared to the MG, PAB, CER, and TRF levels were significantly increased after Acacetin and Mosapride treatment (Figure 5(a)). Compared with the CG, serum TG level was increased, and LDL-C, HDL-C, and TC levels were significantly decreased in the MG. Compared with the MG, serum TG level was decreased, and LDL-C, HDL-C, and TC levels were increased after Acacetin and Mosapride treatment (Figure 5(b)). These results suggested that Acacetin could regulate the protein and lipid metabolism of gastrointestinal motility disorder mice, and the effect of high-dose Acacetin was the best.

### 3.6. Acacetin Regulated Gastrointestinal Inflammation and Apoptosis

**Figure 6:**
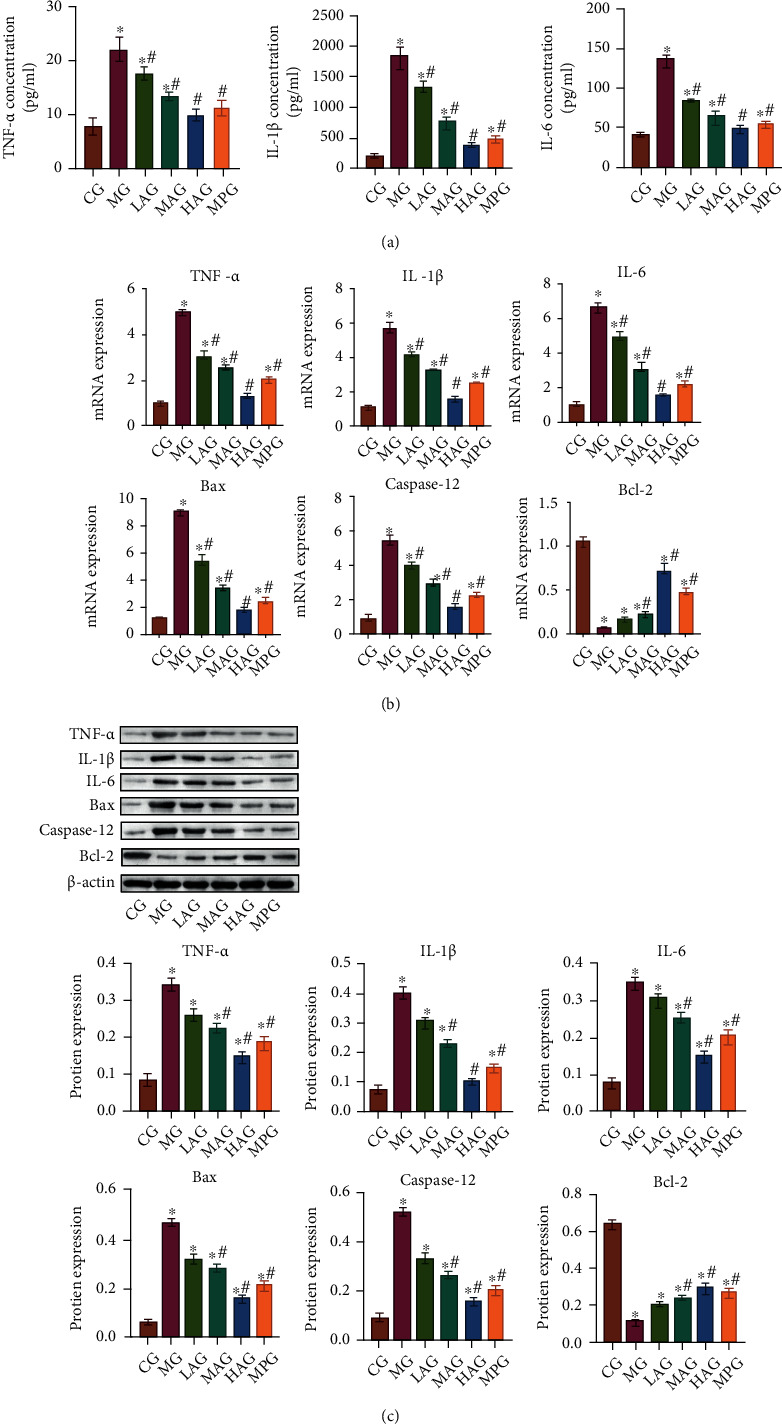
Acacetin regulated gastrointestinal inflammation and apoptosis. (a) The levels of TNF-*α*, IL-1*β*, and IL-6 in serum of mice were determined by ELISA. (b) qRT-PCR was performed to detect the mRNA expressions of inflammatory cytokines TNF-*α*, IL-1*β*, and IL-6 and apoptotic proteins Bcl-2, Bax, and Caspase-12. (c) Western blot was used to detect the protein expressions of inflammatory cytokines TNF-*α*, IL-1*β*, and IL-6 and apoptotic proteins Bcl-2, Bax, and Caspase-12. CG: control group; MG: model group, LAG: low-dose Acacetin group; MAG: middle-dose Acacetin group; HAG: high-dose Acacetin group; MPG: Mosapride group; ^∗^*P* < 0.05 vs. CG; ^#^*P* < 0.05 vs. MG.

To investigate the effects of Acacetin on gastrointestinal inflammation and apoptosis, we first detected the levels of inflammation-related indicators. Compared with the CG, serum levels of TNF-*α*, IL-1*β*, and IL-6 were significantly increased in the MG. Compared with the MG, the levels of TNF-*α*, IL-1*β*, and IL-6 were decreased after Acacetin and Mosapride treatment (Figure 6(a)). The effect of high-dose Acacetin was the best, indicating that Acacetin could inhibit the inflammatory response. Next, we validated the levels of apoptosis-related markers. qRT-PCR and Western blot results showed that, compared with the CG, the expression of Bcl-2 was decreased, the expression of Bax and Caspase-12 was increased, and the expression of proinflammatory cytokines TNF-*α*, IL-1*β*, and IL-6 was also increased in the MG. Compared with the MG, the expression of Bcl-2 was increased, the expression of Bax and Caspase-12 was decreased, and the expression of proinflammatory cytokines TNF-*α*, IL-1*β*, and IL-6 was also decreased after the treatment of Acacetin and Mosapride (Figures 6(b) and 6(c)). These results suggested that Acacetin could regulate gastrointestinal inflammation and apoptosis.

### 3.7. Acacetin Improved Gastrointestinal Motility by Inhibiting the Activation of the PI3K-AKT Signaling Pathway

**Figure 7:**
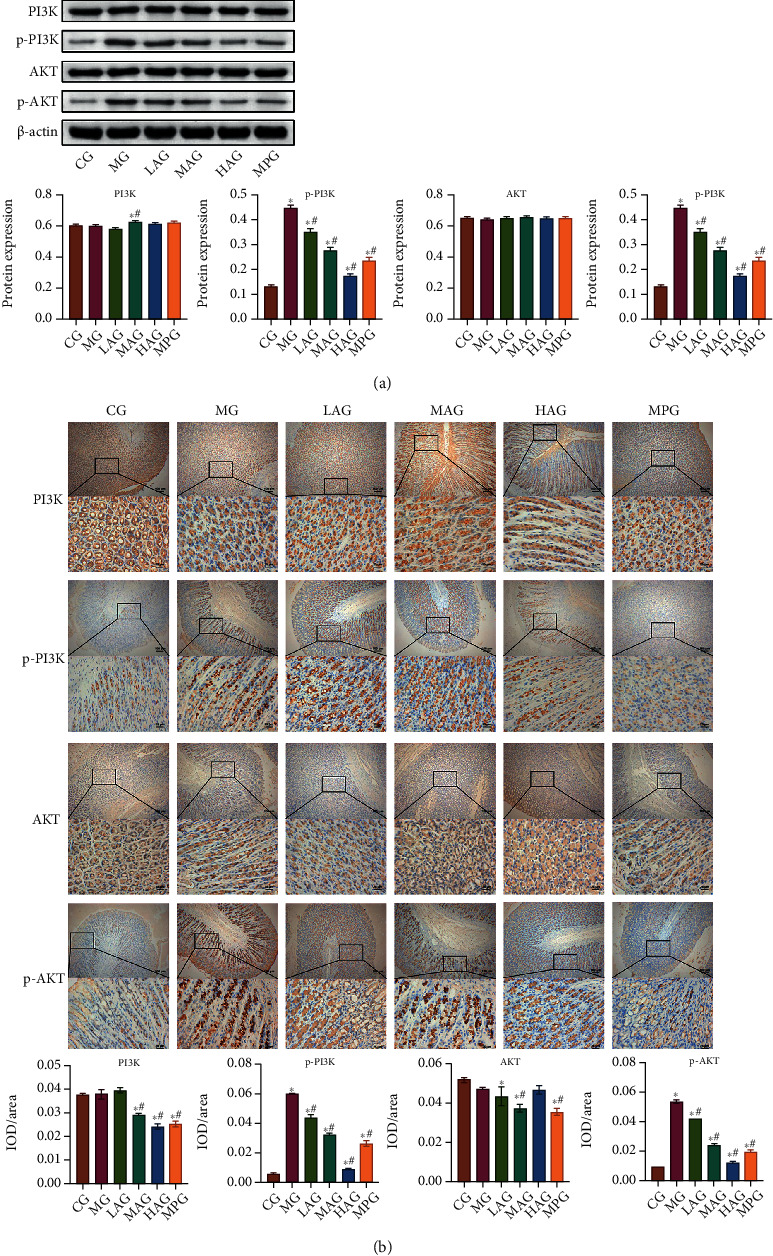
Acacetin improved gastrointestinal motility by inhibiting the activation of the PI3K-AKT signaling pathway. (a) The expression of the PI3K-AKT signaling pathway-related proteins PI3K, p-PI3K, AKT, and p-AKT was detected by Western blot. (b) IHC was used to measure the expression of PI3K, p-PI3K, AKT, and p-AKT in gastric antrum tissues. CG: control group; MG: model group; LAG: low-dose Acacetin group; MAG: middle-dose Acacetin group; HAG: high-dose Acacetin group; MPG: Mosapride group; ^∗^*P* < 0.05 vs. CG; ^#^*P* < 0.05 vs. MG; scale bar = 25 *μ*m; the magnification was 400 times; scale bar = 100 *μ*m; the magnification was 100 times.

Based on the results of KEGG enrichment analysis, we analyzed the role of Acacetin in the regulation of the PI3K-AKT signaling pathway. Western blot and IHC were performed to detect the expression of PI3K-AKT signaling pathway-related proteins PI3K, p-PI3K, AKT, and p-AKT. The results showed that Acacetin and Mosapride improved the expression of p-PI3K and p-AKT. That was, by downregulating PI3K and AKT, the gastrointestinal motility disorders were improved, and the effect of high-dose Acacetin was better (Figures 7(a) and 7(b)). Overall, these data revealed that high-dose Acacetin could improve gastrointestinal motility disorders by inhibiting the activation of the PI3K-AKT signaling pathway.

## 4. Discussion

Gastrointestinal motility disorder is a common gastrointestinal disease that seriously affects life quality. Based on network pharmacological methods, we conducted many experiments. The results showed that high-dose Acacetin could regulate the PI3K-AKT signaling pathway to inhibit gastrointestinal inflammation and apoptosis and regulate protein metabolism and lipid metabolism. It is helpful to regulate the contents of GAS and MTL in mice with gastrointestinal motility disorders and improve gastric emptying ability, which plays an essential role in alleviating gastrointestinal motility disorders.

A variety of hormones play a regulatory role in gastric motility, among which GAS and MTL are the main hormones associated with gastrointestinal motility disorders. GAS is a crucial gastrointestinal hormone that is mainly secreted by G cells [26]. GAS can biaxially regulate gastric acid secretion to keep the gastric acid level in dynamic balance, and the physiological level of GAS could promote gastric emptying [27]. MTL is a protein encoded by the MLNR gene, which can cause the intense contraction of phase III of interdigestive transitional complex movement, promoting gastrointestinal muscle contraction and accelerating gastric emptying [28]. Studies have found that Salsola Collina, an edible plant, can promote gastric emptiness and intestinal propulsion [29]. In the functional gastrointestinal disease with weight loss, after using mirtazapine, the levels of GAS and MTL will be significantly increased [30]. In this study, the levels of GAS and MTL were increased after Acacetin and Mosapride treatment. It suggested that Acacetin could modulate GAS and MTL levels in gastrointestinal motility disorder mice, and high-dose Acacetin has the best effect.

Serum PAB is an effective tool for evaluating malnourished patients [31]. It has been reported that the level of PAB in the study group was significantly higher than that in the control group when parenteral nutrition was given after the gastric cancer surgery [32]. CER is a kind of ferrous oxidase in mammalian plasma and plays a vital role in the iron metabolism [33]. TRF is thought to be the “delivery system” for many beneficial and harmful metal ions to enter cells [34]. It has been reported that patients with inflammatory bowel disease have abnormal lipid metabolism compared with healthy people [35]. In Crohn disease patients, lipid profiles, lipoprotein concentration and composition, and oxidation-antioxidant status are disturbed [36]. We speculated that the abnormality of protein and lipid metabolism might be related to gastrointestinal motility disorders. The results showed that after using Acacetin, the levels of PAB, CER, and TRF were significantly increased, the levels of LDL-C, HDL-C, and TC were increased, and TG level was decreased. These results indicated that high-dose Acacetin could regulate protein metabolism and lipid metabolism in gastrointestinal motility disorder mice, and high-dose Acacetin has the best effect. The analysis of the correlation between nutrient metabolic indexes and disease conditions might be helpful to provide better clinical treatment and improve prognosis.

During the occurrence of gastrointestinal motility disorders, cytokines mediating the inflammatory response in the injury area can secrete inflammatory factors. Acacetin has antioxidant and anti-inflammatory effects [37]. In the study of cerebral ischemia-reperfusion injury, the expression of TNF-*α*, IL-1*β*, and IL-6 was significantly decreased after Acacetin treatment compared with the middle cerebral artery occlusion group [38]. Acacetin can inhibit the increase of TNF-*α*, IL-6, and IL-1*β* in the lipopolysaccharide-stimulated human periodontal ligament cells in a dose-dependent manner and inhibit cell apoptosis [39]. In our study, the levels of TNF-*α*, IL-1*β*, and IL-6 were decreased after using Acacetin, while the expression of Bcl-2 was increased, and the expression of Bax and Caspase-12 was decreased. These results suggested that Acacetin could regulate gastrointestinal inflammation and apoptosis, and high-dose Acacetin has the best effect. As a classical signaling pathway, the PI3K-AKT signaling pathway plays a vital role in the diseases. Caulis Sargentodoxae could regulate the PI3K-AKT signaling pathway to exert anti-inflammatory effects [40]. PI3K could respond to LPS and TNF-*α*, activate AKT, and affect NF-*κ*B to produce antiapoptotic and proinflammatory effects [41]. Our study found that high-dose Acacetin improved gastrointestinal motility disorders by inhibiting the activation of the PI3K-AKT signaling pathway.

However, our study has some limitations. As shown in Table 2, the degree of Acacetin was 64, and the degree of Galangin was 63. The two compounds did not differ obviously in terms of topological parameters. Therefore, we also should experimentally evaluate the therapeutic effect of Galangin. However, due to the limitation of experimental funds, we cannot experimentally evaluate the therapeutic effect of Galangin at present. In the future, we will further investigate the effect of Galangin on gastrointestinal motility disorders and the mechanisms involved and compare the efficacy of Acacetin and Galangin.

In conclusion, our findings suggest that high-dose Acacetin regulates the PI3K-AKT signaling pathway to inhibit gastrointestinal inflammation and apoptosis, regulate protein metabolism and lipid metabolism, and help regulate the levels of GAS and MTL. Acacetin treatment has high efficacy and safety, which could significantly improve the gastrointestinal motility disorder mice, improve the ultrastructure of gastrointestinal tissue, and inhibit the inflammatory response. Our study provides strong experimental support for the clinical treatment of gastrointestinal motility disorders with Acacetin.

## Figures and Tables

**Table 1:** The primers used in this study.

Primer ID	5′-3′
TNF-*α*-F	CCCCTCTATTTATAATTGCACCT
TNF-*α*-R	CTGGTAGTTTAGCTCCGTTT
IL-1*β*-F	CAGCAGCATCTCGACAAGAG
IL-1*β*-R	AAAGAAGGTGCTTGGGTCCT
IL-6-F	TCACTATGAGGTCTACTCGG
IL-6-R	CATATTGCCAGTTCTTCGTA
Caspase-12-F	ATAAAGAGCCAGATATTCTTCGT
Caspase-12-R	TCACCCCTCTCAGTGGTCA
Bax-F	TTGCTACAGGGTTTCATCCAGG
Bax-R	GCTCCAAGGTCAGCTCAGGT
Bcl-2-F	CTGGTGGACAACATCGCTCT
Bcl-2-R	ATAGTTCCACAAAGGCATCCCA
*β*-Actin-F	ACATCCGTAAAGACCTCTATGCC
*β*-Actin-R	TACTCCTGCTTGCTGATCCAC

**Table 2:** The key ingredients in Weishu decoction.

Name	Average shortest path length	Betweenness centrality	Closeness centrality	Degree
Acacetin	2.411523	0.030515	0.414676	64
Galangin	2.427984	0.029059	0.411864	63
Dihydrocapsaicin	2.502058	0.027317	0.399671	56
Matairesinol	2.55144	0.022025	0.391935	51
Albiflorin	2.600823	0.015837	0.384494	42
Imperatorin	2.633745	0.012516	0.379688	38
Formononetin	2.699588	0.008011	0.370427	31
Guanosine	2.716049	0.008303	0.368182	30
Trehalose	2.823045	0.003325	0.354227	20
Chlorogenic acid	2.831276	0.001778	0.353198	17
Guanosine cyclic monophosphate	2.930041	0.000886	0.341292	11
Calycosin-7-O-beta-D-glucoside	2.888889	0.000502	0.346154	8
Loganic acid	2.938272	0.000189	0.340336	5

## Data Availability

All available data information is included in the article.

## Supplementary Materials

Supplementary Materials Supplementary Materials Supplementary Table 1: a total of 15 active components in Weishu Decoction. Supplementary Table 2: a total of 460 target compounds in Weishu Decoction.
