# Supplementary material for: The Key Ingredient Acacetin in Weishu Decoction Alleviates Gastrointestinal Motility Disorder Based on Network Pharmacology Analysis
Source: Mediators Inflamm. 2021 Sep 21;2021:5265444. doi: 10.1155/2021/5265444 (PMC8478590; doi:10.1155/2021/5265444)
Supplement: Supplementary Materials — Supplementary Table 1: a total of 15 active components in Weishu Decoction. Supplementary Table 2: a total of 460 target compounds in Weishu Decoction. [file 5265444.f1.zip › Revised Supplementary Table 1.pdf]

Supplementary Table 1. A total of 15 active components in Weishu Decoction.

MOL ID	Name	MW (Dalton)	AlogP	Hdon (n)	Hacc (n)	OB (%)
MOL002687	Guanosine	283.28	-2.41	6	9	21.43
MOL001955	CHLOROGENIC ACID	354.34	-0.42	6	9	11.93
MOL008698	dihydrocapsaicin	307.48	4.33	2	4	47.07
MOL001927	albiflorin	480.51	-1.33	5	11	12.09
MOL009290	calycosin-7-O-beta-D-glucoside	446.44	0.41	5	10	5.49
MOL000392	FORMONONETIN	268.28	2.58	1	4	69.67
MOL002563	Galangin	270.25	2.04	3	5	45.55
MOL001941	imperatorin	270.3	3.65	0	4	34.55
MOL000526	Trehalose	342.34	-4.26	8	11	2.32
MOL012951	Guanosine cyclic monophosphate	345.24	-1.98	5	11	3.76
MOL000655	loganic acid	376.4	-2.33	6	10	4.92
MOL001955	yl quinic acid (isomer of 831, 83	354.34	-0.42	6	9	11.93
MOL001689	Acacetin	284.28	2.59	2	5	34.97
MOL000433	FA 18:1+3O	441.45	0.01	7	13	68.96
MOL000537	Matairesinol	358.42	3.47	2	6	4.5

MW: molecule weight, ALogP: the value of partition coefficient between octanol and water, Hdon: hydrogen-bond acceptors, OB: Oral bioavailability, BBB: Blood brain barrier, DL: drug-likeness, FASA-, Fractional wa atoms with negative partial charge.

BBB	DL	FASA-
-1.62	0.21	0.23
-1.71	0.33	0.37
0.47	0.19	0.22
-2.19	0.77	0.36
-2.05	0.81	0.27
0.02	0.21	0
-0.09	0.21	0.41
0.92	0.22	0.28
-7	0.24	0.23
-1.96	0.4	0.39
-2.51	0.4	0.26
-1.71	0.33	0.37
-0.05	0.24	0.35
-2.59	0.71	0
-0.23	0.4	0.29

bond donors, Hacc: hydrogen-
ter accessible surface area of all
