# Supplementary material for: The Key Ingredient Acacetin in Weishu Decoction Alleviates Gastrointestinal Motility Disorder Based on Network Pharmacology Analysis
Source: Mediators Inflamm. 2021 Sep 21;2021:5265444. doi: 10.1155/2021/5265444 (PMC8478590; doi:10.1155/2021/5265444)
Supplement: Supplementary Materials — Supplementary Table 1: a total of 15 active components in Weishu Decoction. Supplementary Table 2: a total of 460 target compounds in Weishu Decoction. [file 5265444.f1.zip › Revised Supplementary Table 2.pdf]

Supplementary Table 2. A total of 460 target compounds in Weishu Decoction.

Active component	Node IDs
Acacetin	CYP1B1
Acacetin	CYP19A1
Acacetin	CA7
Acacetin	CA12
Acacetin	CA4
Acacetin	CBR1
Acacetin	ABCC1
Acacetin	ABCB1
Acacetin	TNKS2
Acacetin	TNKS
Acacetin	ESR1
Acacetin	ESR2
Acacetin	HSD17B2
Acacetin	CDK5R1
Acacetin	XDH
Acacetin	CCNB3
Acacetin	ACHE
Acacetin	CDK6
Acacetin	NOX4
Acacetin	AKR1B1
Acacetin	ABCG2
Acacetin	FLT3
Acacetin	HSD17B1
Acacetin	LCK
Acacetin	MAOA
Acacetin	ADORA1
Acacetin	ADORA2A
Acacetin	KIT
Acacetin	OPRD1
Acacetin	SYK
Acacetin	GSK3B
Acacetin	TTR
Acacetin	AKR1B10
Acacetin	ALOX15
Acacetin	ALOX12
Acacetin	CA2
Acacetin	PTPRS
Acacetin	PTGS2
Acacetin	CSNK2A1
Acacetin	CFTR
Acacetin	PLG
Acacetin	CA1
Acacetin	CA9
Acacetin	GRK6
Acacetin	MCL1
Acacetin	PIM1
Acacetin	ARG1
Acacetin	ALOX5
Acacetin	PARP1
Acacetin	APP
Acacetin	KDM4E
Acacetin	CDK1
Acacetin	GLO1
Acacetin	MMP9

Acacetin	MMP2	
Acacetin	MMP12	
Acacetin	CD38	
Acacetin	TOP1	
Acacetin	ADORA3	
Acacetin	PLA2G2A	
Acacetin	NAE1	
Acacetin	TYR	
Acacetin	AMY1A	
Acacetin	AHR	
Acacetin	ESRRA	
Acacetin	SLC22A12	
Acacetin	TERT	
Acacetin	PFKFB3	
Acacetin	AR	
Acacetin	MAOB	
Acacetin	NOS2	
Acacetin	IGF1R	
Acacetin	EGFR	
Acacetin	F2	
Acacetin	ST6GAL1	
Acacetin	SIGMAR1	
Acacetin	BACE1	
Acacetin	CALM1	
Acacetin	CDK2	
Acacetin	CYP1A1	
Acacetin	CYP1A2	
Acacetin	DAPK1	
Acacetin	GPR35	
Acacetin	MPG	
Acacetin	MET	
Acacetin	OPRM1	
Acacetin	BCHE	
Acacetin	AVPR2	
Acacetin	AURKB	
Acacetin	DRD4	
Acacetin	MPO	
Acacetin	PIK3R1	
Acacetin	PYGL	
Acacetin	SRC	
Acacetin	PTK2	
Acacetin	KDR	
Acacetin	MMP13	
Acacetin	MMP3	
Acacetin	CA3	
Acacetin	PLK1	
Acacetin	CDK5	
Acacetin	CCNB1	
Acacetin	CCNB2	
dihydrocapsaicin	TRPV1	
dihydrocapsaicin	PTGS1	
dihydrocapsaicin	CYP1A2	CNR1
dihydrocapsaicin	ACHE	SLC6A11
dihydrocapsaicin	BCHE	SLC6A13
dihydrocapsaicin	SIGMAR1	CHRM4
dihydrocapsaicin	CNR1	DRD3

dihydrocapsaicin	SLC6A11	HDAC8
dihydrocapsaicin	SLC6A13	HDAC1
dihydrocapsaicin	CHRM4	HDAC6
dihydrocapsaicin	DRD3	CNR2
dihydrocapsaicin	HDAC8	ROCK2
dihydrocapsaicin	HDAC1	OPRK1
dihydrocapsaicin	HDAC6	SLC6A4
dihydrocapsaicin	CNR2	HDAC3
dihydrocapsaicin	ROCK2	CHRM2
dihydrocapsaicin	OPRK1	HRH2
dihydrocapsaicin	SLC6A4	MME
dihydrocapsaicin	HDAC3	ADRA2A
dihydrocapsaicin	CHRM2	ADRA2C
dihydrocapsaicin	AR	HRH1
dihydrocapsaicin	HRH2	HDAC10
dihydrocapsaicin	MME	EPHX2
dihydrocapsaicin	ADRA2A	HDAC11
dihydrocapsaicin	ADRA2C	SCN9A
dihydrocapsaicin	HRH1	CDC25A
dihydrocapsaicin	HDAC10	CDC25B
dihydrocapsaicin	EPHX2	DRD2
dihydrocapsaicin	HDAC11	HTR2A
dihydrocapsaicin	SCN9A	CHEK1
dihydrocapsaicin	CDC25A	RAF1
dihydrocapsaicin	CDC25B	FGFR1
dihydrocapsaicin	DRD2	BRAF
dihydrocapsaicin	HTR2A	CHRM1
dihydrocapsaicin	CHEK1	SLC6A2
dihydrocapsaicin	RAF1	MAPKAPK2
dihydrocapsaicin	FGFR1	NPY1R
dihydrocapsaicin	BRAF	CHRM5
dihydrocapsaicin	DRD4	CHRM3
dihydrocapsaicin	CHRM1	LIPG
dihydrocapsaicin	SLC6A2	ADRA2B
dihydrocapsaicin	MAPKAPK2	DYRK1A
dihydrocapsaicin	NPY1R	HSD17B3
dihydrocapsaicin	CHRM5	PLAU
dihydrocapsaicin	CHRM3	NEK1
dihydrocapsaicin	LIPG	YES1
dihydrocapsaicin	ADRA2B	EPHB4
dihydrocapsaicin	CDK5R1	HTR2B
dihydrocapsaicin	DYRK1A	MMP1
dihydrocapsaicin	HSD17B3	MTOR
dihydrocapsaicin	PLAU	PIK3CG
dihydrocapsaicin	NEK1	PIK3CA
dihydrocapsaicin	YES1	WEE1
dihydrocapsaicin	EPHB4	CXCR3
dihydrocapsaicin	HTR2B	KCNH2
dihydrocapsaicin	MMP9	SPHK2
dihydrocapsaicin	MMP1	SPHK1
dihydrocapsaicin	MMP2	FAAH
dihydrocapsaicin	FLT3	RET
dihydrocapsaicin	MTOR	CCR8
dihydrocapsaicin	PIK3CG	RELA
dihydrocapsaicin	PIK3CA	DPP7
dihydrocapsaicin	WEE1	ERN1

dihydrocapsaicin	CXCR3	DUSP3
dihydrocapsaicin	KCNH2	HSP90AA1
dihydrocapsaicin	SPHK2	CDC7
dihydrocapsaicin	SPHK1	ODC1
dihydrocapsaicin	FAAH	SGK1
dihydrocapsaicin	RET	MYLK
dihydrocapsaicin	CCR8	LIMK1
dihydrocapsaicin	RELA	CDC42BPA
dihydrocapsaicin	DPP7	TNK2
dihydrocapsaicin	ERN1	DMPK
dihydrocapsaicin	DUSP3	MMP7
dihydrocapsaicin	HSP90AA1	PKN2
dihydrocapsaicin	CDC7	TLR9
dihydrocapsaicin	ODC1	HTR1A
dihydrocapsaicin	SGK1	MPEG1
dihydrocapsaicin	MYLK	KAT2B
dihydrocapsaicin	LIMK1	F9
dihydrocapsaicin	CDC42BPA	PDGFRB
dihydrocapsaicin	TNK2	SCD
dihydrocapsaicin	DMPK	PIK3CD
dihydrocapsaicin	MMP13	PIK3CB
dihydrocapsaicin	MMP7	STK17B
dihydrocapsaicin	PKN2	MAP4K4
dihydrocapsaicin	TLR9	TYMS
dihydrocapsaicin	HTR1A	DHFR
dihydrocapsaicin	MPEG1	SLC19A1
dihydrocapsaicin	KAT2B	SLC46A1
dihydrocapsaicin	F9	FOLR1
dihydrocapsaicin	ADORA3	FPGS
dihydrocapsaicin	PDGFRB	FOLR2
dihydrocapsaicin	KIT	GART
dihydrocapsaicin	PFKFB3	ATIC
dihydrocapsaicin	SCD	SLC5A1
dihydrocapsaicin	PIK3CD	IL2
dihydrocapsaicin	PIK3CB	ALDH2
dihydrocapsaicin	STK17B	TBXAS1
dihydrocapsaicin	MAP4K4	MGAM
dihydrocapsaicin	CDK5	HTR2C
FA 18_1+3O	TYMS	ESRRB
FA 18_1+3O	DHFR	MIF
FA 18_1+3O	SLC19A1	PPARA
FA 18_1+3O	SLC46A1	PON1
FA 18_1+3O	FOLR1	CA6
FA 18_1+3O	HDAC8	CA14
FA 18_1+3O	FPGS	CA13
FA 18_1+3O	FOLR2	CA5B
FA 18_1+3O	HDAC6	CA5A
FA 18_1+3O	HDAC1	DHODH
FA 18_1+3O	GART	STS
FA 18_1+3O	ATIC	ERCC5
FA 18_1+3O	NOS2	FEN1
FA 18_1+3O	SLC5A1	PTPN1
FORMONONETIN	IL2	ALK
FORMONONETIN	CYP19A1	AXL
FORMONONETIN	ALDH2	MAPT
FORMONONETIN	ESR1	TOP2A

FORMONONETIN	CA12	INSR
FORMONONETIN	ADORA1	APEX1
FORMONONETIN	ADORA2A	IKBKB
FORMONONETIN	TBXAS1	NTRK2
FORMONONETIN	MAOA	CXCR1
FORMONONETIN	MGAM	PKN1
FORMONONETIN	HTR2A	NEK2
FORMONONETIN	HTR2C	CAMK2B
FORMONONETIN	ESRRA	AKT1
FORMONONETIN	ESRRB	NEK6
FORMONONETIN	ABCG2	KCNA3
FORMONONETIN	CA7	GABRB3
FORMONONETIN	HSD17B2	GABRA2
FORMONONETIN	CA4	MAPK14
FORMONONETIN	CBR1	ADORA2B
FORMONONETIN	SLC6A2	NTRK1
FORMONONETIN	ALOX12	PDE10A
FORMONONETIN	EGFR	ADRA1B
FORMONONETIN	TYR	JAK2
FORMONONETIN	XDH	HDAC2
FORMONONETIN	PFKFB3	CSNK1D
FORMONONETIN	ESR2	FNTA
FORMONONETIN	PTPRS	PGGT1B
FORMONONETIN	HSD17B1	CCNE2
FORMONONETIN	ABCB1	TAAR1
FORMONONETIN	ALOX15	PTGER1
FORMONONETIN	MIF	MCHR1
FORMONONETIN	PPARA	GRM5
FORMONONETIN	PTGS1	PRKDC
FORMONONETIN	TLR9	PDE5A
FORMONONETIN	MAOB	BRS3
FORMONONETIN	CA2	TGFBR1
FORMONONETIN	CA1	PTK2B
FORMONONETIN	NOX4	GYS1
FORMONONETIN	PON1	GSK3A
FORMONONETIN	TNKS2	CLK4
FORMONONETIN	TNKS	HSF1
FORMONONETIN	ACHE	PDE8B
FORMONONETIN	CA3	PGR
FORMONONETIN	CA6	MAPK8
FORMONONETIN	CA14	CSNK1G1
FORMONONETIN	CA13	CCR2
FORMONONETIN	CA5B	PPOX
FORMONONETIN	CA5A	ADRA1A
FORMONONETIN	DHODH	PTGER4
FORMONONETIN	STS	PTGER2
FORMONONETIN	ERCC5	PTGER3
FORMONONETIN	FEN1	HCRTR2
FORMONONETIN	RAF1	HCRTR1
FORMONONETIN	CA9	SRD5A1
FORMONONETIN	PTPN1	MAP2K1
FORMONONETIN	BACE1	NAAA
Galangin	BCHE	PTAFR
Galangin	XDH	HRH3
Galangin	ACHE	JAK1
Galangin	ADORA1	TYK2

Galangin	CA7	GABRA3
Galangin	ADORA2A	GABRG2
Galangin	ADORA3	FNTB
Galangin	CA12	GABRA1
Galangin	CA4	GABRA5
Galangin	ABCB1	CCNE1
Galangin	CYP1B1	HSPA8
Galangin	AKR1B1	HSPA5
Galangin	CA2	ADK
Galangin	ABCG2	DPP4
Galangin	HSD17B2	ADA
Galangin	HSD17B1	EIF4E
Galangin	ALOX15	GAPDH
Galangin	ESR2	HPRT1
Galangin	NOX4	PNP
Galangin	FLT3	CDC42
Galangin	ABCC1	RAC1
Galangin	ALOX5	AHCY
Galangin	PIM1	POLA1
Galangin	CSNK2A1	CDA
Galangin	EGFR	TYMP
Galangin	ESRRA	GAA
Galangin	TYR	OGA
Galangin	IGF1R	TK1
Galangin	AURKB	CCND1
Galangin	KDR	MAPK1
Galangin	PLK1	EHMT1
Galangin	MET	EHMT2
Galangin	ALK	FUCA1
Galangin	AXL	GRK1
Galangin	CYP19A1	GBA
Galangin	CA1	SETD7
Galangin	CA9	HK2
Galangin	AHR	HK1
Galangin	KDM4E	DAO
Galangin	CDK1	SRM
Galangin	ALOX12	PDCD4
Galangin	ESR1	CDK4
Galangin	MAOA	SLC37A4
Galangin	SYK	PRKCD
Galangin	GSK3B	PRKCA
Galangin	CDK5R1	NEU4
Galangin	CCNB3	PDE4D
Galangin	CDK6	PDE9A
Galangin	CBR1	PDE1B
Galangin	GRK6	CASP3
Galangin	MAPT	NEU3
Galangin	TOP2A	NEU2
Galangin	INSR	ENGASE
Galangin	MYLK	ECE1
Galangin	APEX1	ELANE
Galangin	PFKFB3	CASP6
Galangin	TNKS2	CASP7
Galangin	TNKS	CASP8
Galangin	SLC22A12	CASP1
Galangin	PTPRS	CASP2

Galangin	PTGS2	EGLN1
Galangin	TTR	ADAMTS5
Galangin	CFTR	FTO
Galangin	AKR1B10	DNMT3B
Galangin	IKBKB	TREH
Galangin	NTRK2	LGALS3
Galangin	PIK3CG	LGALS9
Galangin	TERT	SELP
Galangin	GLO1	SLC29A1
Galangin	MMP9	PPM1A
Galangin	MMP2	MMP8
Galangin	SRC	LGALS7
Galangin	PARP1	VEGFA
Galangin	F2	FGF1
Galangin	NAE1	FGF2
Galangin	BACE1	FDFT1
Galangin	LCK	SERPINE1
Galangin	GPR35	SQLE
Galangin	DAPK1	MGMT
Galangin	MPG	AGTR1
Galangin	CDK2	HRAS
Galangin	ARG1	SLC28A2
Galangin	MCL1	PRKCG
Galangin	CXCR1	PRKCB
Galangin	AVPR2	PRKCE
Galangin	DRD4	PRKCH
Galangin	MPO	ADAM17
Galangin	PIK3R1	SLC28A3
Galangin	PYGL	IMPDH1
Galangin	PTK2	MAP3K7
Galangin	MMP13	ABL1
Galangin	MMP3	EPHA2
Galangin	CA3	MAP3K9
Galangin	CA6	AURKA
Galangin	PKN1	BTK
Galangin	CA14	SSTR2
Galangin	NEK2	SSTR4
Galangin	CAMK2B	SSTR1
Galangin	AKT1	SSTR3
Galangin	NEK6	EIF4H
Galangin	CDK5	PABPC1
Galangin	CCNB1	TNF
Galangin	CCNB2	PSEN2
imperatorin	MAOA	HPSE
imperatorin	ACHE	LGALS4
imperatorin	KCNA3	LGALS8
imperatorin	BACE1	FOLH1
imperatorin	CYP1A2	DRD1
imperatorin	GABRB3	ADRA1D
imperatorin	GABRA2	CYP2D6
imperatorin	CYP19A1	HTR6
imperatorin	MAPK14	HTR1B
imperatorin	ADORA2B	RORC
imperatorin	NOS2	STAT3
imperatorin	ADORA1	PSENEN
imperatorin	NTRK1	NCSTN

imperatorin	PDE10A	APH1A
imperatorin	ADRA2A	PSEN1
imperatorin	ADRA2C	APH1B
imperatorin	ADRA2B	RNASE2
imperatorin	ADRA1B	RNASE1
imperatorin	JAK2	RARS
imperatorin	HDAC3	POLB
imperatorin	HDAC2	FBP1
imperatorin	HDAC1	FHIT
imperatorin	CSNK1D	RNASEL
imperatorin	FNTA	QARS
imperatorin	PGGT1B	DTYMK
imperatorin	CCNE2	HSPA1A
imperatorin	TAAR1	DNPH1
imperatorin	PTGER1	HLCS
imperatorin	MTOR	P2RX1
imperatorin	PIK3CD	P2RY6
imperatorin	PIK3CB	OGT
imperatorin	PIK3CG	P2RY1
imperatorin	MCHR1	P2RY11
imperatorin	PIK3CA	PARG
imperatorin	GRM5	SELL
imperatorin	PRKDC	NAALAD2
imperatorin	PDE5A	SHBG
imperatorin	BRS3	LYPLA1
imperatorin	MAOB	LYPLA2
imperatorin	TGFBR1	SLC6A3
imperatorin	PTK2B	GCGR
imperatorin	FAAH	HSD11B1
imperatorin	GYS1	UPP1
imperatorin	GSK3A	QDPR
imperatorin	CLK4	DNM1
imperatorin	HSF1	CLK1
imperatorin	PDE8B	DYRK1B
imperatorin	DYRK1A	PTGFR
imperatorin	PGR	PIM2
imperatorin	MAPK8	NR3C1
imperatorin	MAPKAPK2	ERBB2
imperatorin	CSNK1G1	HTR7
imperatorin	CCR2	PRKCZ
imperatorin	PPOX	CAPN1
imperatorin	PIM1	CHRNA4
imperatorin	ADRA1A	AKR1C3
imperatorin	CA9	PDK1
imperatorin	PTGER4	PITRM1
imperatorin	PTGER2	MMP14
imperatorin	BCHE	PPARG
imperatorin	PTGER3	ITK
imperatorin	HCRTR2	PNMT
imperatorin	HCRTR1	LNPEP
imperatorin	CNR1	JAK3
imperatorin	CNR2	BLK
imperatorin	SRD5A1	CDC25C
imperatorin	PTGS1	CSK
imperatorin	PTGS2	EPHB2
imperatorin	MAP2K1	PLA2G7

imperatorin	NAAA	BMX
imperatorin	CA12	LYN
imperatorin	FLT3	EPHA5
imperatorin	PTAFR	EPHA4
imperatorin	HRH3	TXK
imperatorin	JAK1	FGR
imperatorin	TYK2	EPHA6
imperatorin	GABRA3	PTK6
imperatorin	GABRG2	EPHB3
imperatorin	FNTB	EPHA3
imperatorin	CDK2	TYRO3
imperatorin	GABRA1	COQ8B
imperatorin	GABRA5	EPHA1
imperatorin	CCNE1	CHRNB2
